# Discrete choice experiment for dyadic data collection: eliciting preferences of couple-based smoking cessation interventions

**DOI:** 10.4069/whn.2024.03.08.1

**Published:** 2024-03-29

**Authors:** Seung Hee Choi, Thomas Templin

**Affiliations:** College of Nursing, Wayne State University, USA

## Introduction

A discrete choice experiment (DCE) is a validated method of eliciting preferences regarding healthcare services and health behavior interventions, including smoking cessation programs. This approach has been widely used within the field of health economics [1,2]. Grounded in economic theory, DCEs posit that any product or service can be defined by its attributes along with the levels, or subcategories, of those attributes. Additionally, these experiments assume that individuals’ choices regarding a service are influenced by the degree to which they value these attributes and levels [3]. Compared to traditional ratings or ranking scales, DCEs have been shown to more accurately reflect decision-making processes and better clarify potential trade-offs [1].

**Figure 1.**
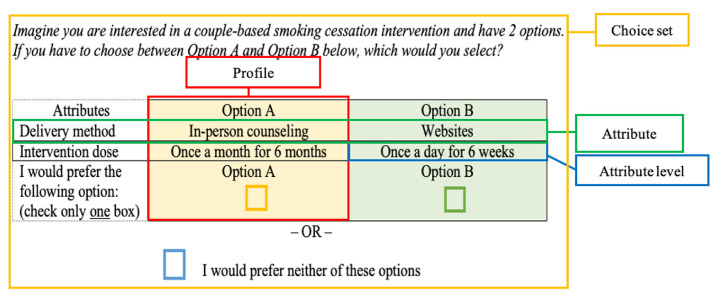
Choice set, attributes, attribute levels, and profiles.

A DCE survey consists of several choice sets, each containing two or more profiles and a potential opt-out option (Figure 1). Each profile includes a specific combination of attribute levels representing a hypothetical intervention. Participants evaluate each choice set and select their preferred option. These answers provide insights into the respondent’s perceptions of the benefits and trade-offs among attributes [3]. For instance, if there is a choice between smoking cessation interventions with two attributes (delivery method and intervention dose), the attribute levels for the former might include in-person counseling and mobile/web applications, while the levels for the latter may be daily sessions over 6 weeks and monthly sessions over 6 months. Each choice set then presents a combination of these attribute levels; for example, Option A might offer in-person counseling in the form of monthly sessions for 6 months, whereas Option B could provide a web-based program with daily sessions for 6 weeks. Ultimately, individuals make an informed decision between Option A or B, based on their perceived value of each (Figure 1).

The use of DCEs is gaining popularity in the literature on smoking cessation, particularly in designing interventions tailored to specific populations. For instance, Katz et al. [4] employed a DCE to determine veterans’ preferences regarding smoking cessation interventions and nicotine replacement therapies (NRTs). Similarly, Salloum et al. [5] conducted a DCE among 191 university students in Lebanon to evaluate their willingness to make trade-offs concerning health risks, distance to treatment facilities, treatment costs, and types of treatment when choosing NRTs. Additionally, DCEs have helped shape tobacco control policies. Pesko and colleagues utilized a DCE of 1,200 adult smokers to assess the potential impact of regulations on electronic nicotine delivery systems (ENDS). Their findings suggest that imposing higher taxes and implementing severe warning labels on ENDS may discourage their use [6].

Despite the widespread utilization of DCEs in the tobacco control literature, no prior study has employed this methodology to explore the preferences of smoker couples for couple-based smoking cessation interventions, nor to understand their desires for partner support (framed as the smoker’s support “demand” and the partner’s “supply”) through the collection of dyadic data. Here, “smoker couples” are married or cohabiting pairs in which at least one partner smokes. For these couples, positive, emotional, and instrumental support from the partner, along with collaborative messages, have been associated with successful cessation [7,8]. Couple-based interventions that include partners in the process have been shown to enhance social support and encourage behavioral changes, such as reducing problem drinking [9] and increasing physical activity [10]. Partner support can act as a buffer against stress, boost motivation and social pressure to change behavior, and foster effective coping strategies [11]. Thus, couple-based smoking cessation interventions are highly promising. Despite the demonstrated success of couple-based interventions for other health behaviors [9,10], a recent review of 11 smoking cessation studies found limited evidence supporting the effectiveness of these interventions. In most of the included studies, long-term quit rates were low, and the interventions did not lead to increased partner support [12]. If interventions overly rely on changes in partner support to satisfy the smoker’s “demand” without considering the partner’s understanding of and willingness to adopt the support role (“supply”), then partner support may become more of a hindrance than a help [13,14]. Gaining deeper in­sight into participants’ preferences for couple-based interven­tions is essential for enhancing the effectiveness of these pro­grams and fostering effective partner support. 

To understand the preferences of smoker couples regarding the format and content of couple-based smoking cessation interventions, we conducted an online DCE survey study. We also evaluated their preferences for partner support in smoking cessation, examining both the perspectives of the smoker’s “demand“ and the partner’s “supply“. In this article, we detail our procedures, including (1) the development of the survey and (2) the collection of dyadic data during individual online meetings. During these sessions, couples engaged in a think-aloud method, verbalizing their thoughts step by step as they completed the survey. This approach has been found effective in exploring the cognitive processes behind decision-making and improving the validity of DCE data [15]. Here, we present preliminary findings on the preferences for the format, content, and partner support of the described interventions, with a full report available elsewhere (unpublished data).

## Methods

Ethics statement: The Institutional Review Board at Wayne State University exempted this study from human participant review (IRB-20-02-1856).

### Study design

An online DCE survey was designed. The survey was individually administered to smoker couples during online interviews, which were conducted via Zoom (https://zoom.us) and supplemented by the incorporation of the think-aloud technique. A couple-based smoking cessation intervention was operationalized as one involving a partner-support element intended to promote smoking cessation [16]. Partner support was defined as any behavior perceived by either the provider of support (the partner) or the recipient (the smoker) to aid in the cessation of smoking [17].

### Survey development

A two-part DCE survey was developed specifically for this study. Part 1 addressed the format and content of couple-based smoking cessation interventions. Part 2 assessed the type of partner support desired by the smokers (Part 2A) and the partners’ willingness and comprehension regarding providing support (Part 2B). For Parts 1 and 2, 6 two-level attributes were identified each, drawing from the existing literature on smoking cessation interventions and partner support in this context. The attributes in Part 1 included convenience, delivery method, partner involvement, topics, duration and frequency of treatment, and interaction style (Table 1). The attributes of Part 2 encompassed the categories of partner support, positive versus negative support, decision-making, presence of partner involvement, strategies versus habits, and frequency of partner support (Table 2).

Both partners selected between two hypothetical scenarios concerning the format and content of a couple-based smoking cessation intervention. They jointly completed Part 1, and then individually filled out Part 2. In Part 2A, primary smokers were presented with scenarios that varied in desired partner support. In Part 2B, partners were given scenarios that differed in the ways they could support the primary smokers. Part 2A and Part 2B featured the same attributes and levels but were tailored to the perspective of each partner. For example, regarding positive versus negative partner support, primary smokers completing Part 2A were shown the statement “Negative support from my partner helps me more to quit smoking,” while partners working through Part 2B saw “Negative support helps my partner more to quit smoking.” In couples with only one partner who smoked, that individual was considered the primary smoker and completed both Part 1 and Part 2A as such, while the nonsmoking partner completed Part 1 and Part 2B. For couples in which both partners smoked, the individual who filled out the screening survey and contacted the research team was deemed the primary smoker. This person answered questions regarding the smoker’s perspective, or demand (Part 1 and Part 2A), while their smoking partner responded to questions from the partner’s viewpoint, or supply (Part 1 and Part 2B).

**Figure 2.**
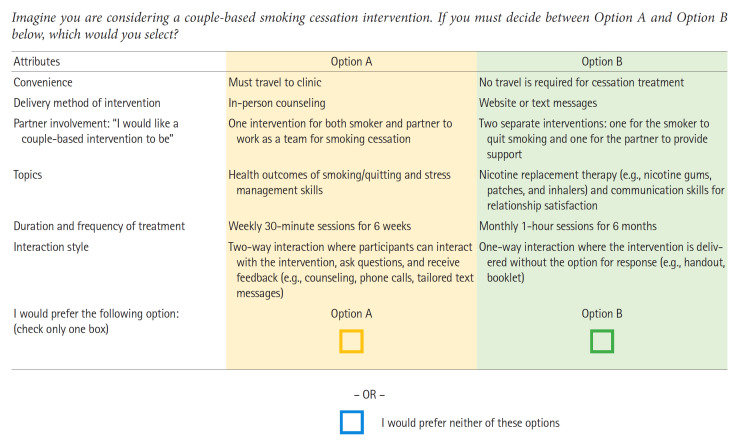
Sample choice set from the discrete choice experiment regarding the format and content of a couple-based smoking cessation intervention.

These differing choice sets and profiles represented a distinction from traditional surveys, in which all participants typically answer the same set of questions. In the present research, each survey attribute can be compared to a factor in a factorial analysis of variance design. A survey with 6 dichotomous attributes represents a 2^6^ factorial design, amounting to 64 cells. However, it would be impractical to ask participants to evaluate 64 combinations. Therefore, a DCE employs a modified fractional factorial design to reduce this number. For this study, the Choice Designs platform within JMP Pro 14 (SAS Institute, Cary, NC, USA) [18], a statistical software package, was used to generate the combinations of attributes and their levels, which were then used to construct the profiles and choice sets. Each choice set consisted of a pair of profiles, with 8 choice sets each in Part 1 and Part 2. To better reflect real-world decision-making regarding interventions, an opt-out option was included. This allowed participants to select “Option A,” “Option B,” or “Neither Option A nor Option B.” The choice sets were designed to be comprehensible at a ninth-grade reading level. Each survey was individually uploaded to Qualtrics, an online survey platform (https://www.qualtrics.com). Figure 2 presents an example of a choice set.

### Collection of dyadic data during individual online meetings

#### Participants and procedures

Smoker couples were recruited through various channels, including community organizations and Facebook ads. To be eligible for inclusion, individuals had to (a) be at least 18 years old, (b) have been married to or living as married with an partner for a minimum of 6 months, and (c) have access to a computer or tablet with an internet connection. Additionally, at least one partner had to (d) be a current smoker and (e) respond positively to the question, “Are you currently attempting to quit or considering quitting smoking within the next 6 months?” The exclusion criteria encompassed individuals who (a) could not understand spoken or written English or (b) were significantly cognitively impaired.

**Figure 3.**
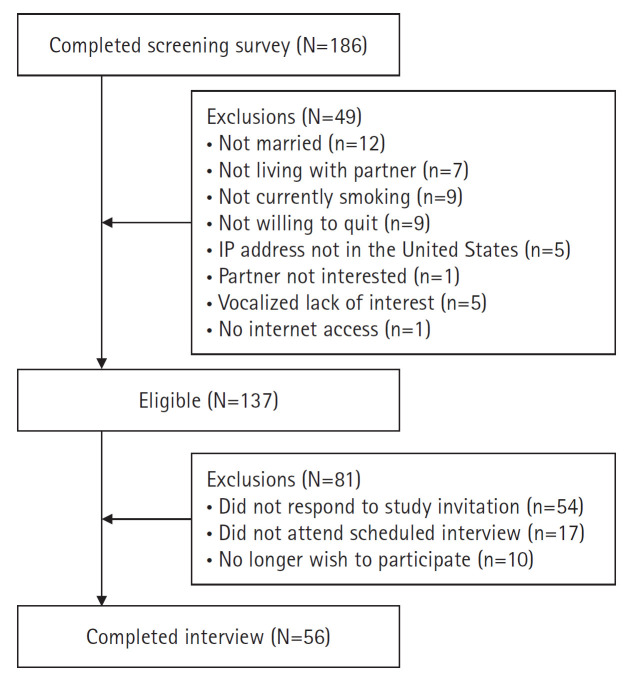
Flowchart of participant recruitment.

Among the 186 responses received from the screening survey, 73.7% of eligible respondents (n=137) were invited to participate in the study. Of these 137 invited couples, 81 did not respond to the invitation, failed to attend, or chose not to participate, resulting in a final sample of 56 smoker couples (Figure 3). Detailed participant information is presented elsewhere (unpublished data). In brief, most primary smokers were male (n=44, 78.6%) and non-Hispanic Black or African American (n=41, 73.2%), with their partners predominantly female (n=44, 78.6%) and similarly non-Hispanic Black or African American (n=41, 73.2%). The mean age of the primary smokers was 32.1 (±8.2) years, while for their partners, it was 29.6 (±7.9) years. Nearly half of the couples (n=24, 42.8%) had an annual income of US $59,999 or less, and the average duration of marriage or cohabitation was 6.3 (±5.5) years. Over 50% of the couples contained only one smoking partner (n=32 couples, 57.1%), while the remainder were dual-smoker couples (n=24, 42.9%). Nearly half of the primary smokers began smoking before the age of 17, and the average duration of smoking was 8.05 (±12.7) years. A total of 23 primary smokers (41.1%) exhibited high nicotine dependence (Fagerstrom Test for Nicotine Dependence ≥6), with a mean score of 4.8 (±2.0). After completing the survey, participants received a US $40 Amazon gift card as a token of appreciation for their participation.

#### Individual online meetings

Eligible and consenting participants were invited to individual Zoom meetings with research staff. Here, participants engaged with a practice choice set (related to house-buying preferences) before proceeding to the study choice sets, and they received explanations about the attributes and their respective levels prior to making their selections. In Part 1, participants were encouraged to engage in discussion and make decisions collaboratively. Additionally, they were asked to share their screens throughout the session to allow research staff to promote the think-aloud method. If participants neglected to verbalize their thoughts using this method, they were gently reminded by the research staff after completing two or three choice sets. Staff also monitored participants for any indications of cognitive fatigue, such as frustration, irritation, or boredom.

All participants successfully completed the DCE survey under this protocol. The average duration of the interviews was 59.55 (±19.29) minutes, with a range of 20.17 to 124.09 minutes. The two most common reasons for interviews exceeding 60 minutes were technical difficulties, including internet connectivity problems and device malfunctions. No respondents exhibited the abovementioned signs of cognitive fatigue during the decision-making process.

#### Think-aloud method

All participants employed the think-aloud method, which facilitated an in-depth understanding of their preferences regarding smoking cessation interventions and partner support. This method also heightened participants’ awareness of their choices, thereby supporting the validity of the DCE data [15]. Furthermore, the think-aloud dialogue provided a platform for smoker couples to engage in discussions about smoking cessation interventions with their partners and to share their preferences. For instance, one participant said, “*I don’t think that [the duration/frequency of treatment] is super important to me, what about you?*” (Couple ID 6). Another remarked, “*I like the nicotine replacement therapy, what do you think?*” (Couple ID 8).

The following two examples illustrate constructive dialogues between partners during decision-making.

Partner A: “*I don’t think [partner involvement] is a high priority. Do you want to be involved?*”

Partner B: “*Yeah, I don’t think that’s necessarily important.*”

Partner A: “*Um, what about willingness to support?*”

Partner B: “*I mean, either way, the partner is going to support.*”

Partner A: “*Have you made up your mind? Well, I think Option A is more important to me. What about you? I’m asking for your opinion*.” (Couple ID 6)

Partner A: “*Two-way interaction where you can have interactive conversations with the intervention, ask questions, get feedback...I like that better. [Don’t] you think?*”

Partner B: “*…you are the one who is still smoking, so whatever benefits you more…so, we [choose] B for this one*.” (Couple ID 8)

After completing several choice sets, couples became familiar with each other’s preferences and were able to make decisions more swiftly. For instance, one partner remarked, “*Again, no travel. We don’t even have to [discuss] that. I already know what he’s going to say.*” (Couple ID 10)

### Statistical analysis

Both partners in all 56 enrolled smoker couples were included in the analysis. This sample size is considered appropriate for exploratory research, as indicated by Orme (2019), who suggests that DCEs include 30 to 60 participants [19]. The DCE data were analyzed using conditional logistic regression models to identify the optimal profiles for smoking cessation interventions targeted at couples [18]. The methodology incorporated a “no-response” option. To adjust for multiple comparisons, Firth bias-adjusted estimates were applied to the *p*-values. All quantitative data were analyzed with SAS 9.4 (SAS Institute) and JMP Pro 14. The think-aloud data were captured on video, transcribed, and analyzed through meticulous line-by-line review.

### Preliminary discrete choice experiment results

Preliminary DCE data revealed six attributes favored by smoker couples in the context of this study. These couples preferred interventions that engaged both partners, were interactive and personalized, encouraged positive support and open discussions between partners, and involved the smoker’s partner in the cessation process. Additionally, smokers expressed a preference for receiving empathy, love, and trust from their partners (Table 3).

## Conclusion

Our study is the first to explore preferences for couple-based smoking cessation interventions and partner support from the perspectives of both partners, utilizing an online DCE survey administered during individual online meetings. The paper provides a detailed account of the development of the DCE survey and the dyadic data collection process employed during these meetings. We found the survey to be a valid and feasible method for gathering dyadic data on the partners’ preferences. Furthermore, the think-aloud technique offered not only a deeper understanding of the participants’ choices but also a valuable opportunity for the partners to reciprocally express their preferences and expectations of partner support. This discussion and collaboration enhanced the validity of the DCE data. However, using the think-aloud technique may also increase the cognitive burden on participants [20]. Therefore, researchers should weigh the advantages and disadvantages of this method when considering its incorporation into their studies.

Despite the increasing popularity and benefits of DCEs, researchers must also be mindful of their limitations and challenges, which include complex survey design, intricate statistical analyses, and the potential for cognitive fatigue among participants. To navigate the survey design and statistical intricacies of DCEs, researchers should seek guidance from experts in the field and consider collaboration. To lessen the cognitive burden on participants, it is essential to employ straightforward language, offer adequate explanations of tasks, and restrict the number of attributes and choice sets. In our study, we introduced a sample choice set (related to purchasing a house) before the study sets and provided clear definitions for each attribute. Consequently, all participants were able to complete the DCE survey without any reports of cognitive fatigue.

This study represents a unique contribution to nursing research through the use of DCE methodology in the design of complex behavioral interventions. Conducted by nurse scientists, this approach enhanced our understanding of couples’ preferences regarding smoking cessation interventions and partner support. Moreover, our findings provide guidance for the development of future couple-based smoking cessation programs. These programs can be customized to meet the specific needs of couples in terms of intervention format and content, as well as the nature of support given and received. Such tailoring is anticipated to promote effective partner support and increase the likelihood of successful smoking cessation among couples with at least one smoker.

## Figures and Tables

**Table 1.** Six attributes and levels pertaining to the format and content of a couple-based smoking cessation intervention (Part 1)

Attribute	Levels
Convenience	1. Must travel to clinic
	2. No travel required for cessation treatment
Delivery method of intervention	1. In-person counseling
	2. Website or text messages
Partner involvement: “I would like a couple-based intervention to be:”	1. Two separate interventions: one for the smoker to quit smoking and one for the partner to provide support
	2. One intervention for both smoker and partner to work as a team for smoking cessation
Topics	1. Health outcomes of smoking/quitting & stress management skills
	2. Nicotine replacement therapy (e.g., nicotine gums, patches, and inhalers) and communication skills for relationship satisfaction
Duration and frequency of treatment	1. Weekly 30-minute sessions for 6 weeks
	2. Monthly 1-hour sessions for 6 months
Interaction style	1. One-way interaction where the intervention is delivered without the option for response (e.g., handout, booklet)
	2. Two-way interaction where participants can interact with the intervention, ask questions, and receive feedback (e.g., counseling, phone calls, tailored text messages)

**Table 2.** Six attributes and levels pertaining to partner support (Part 2)

Attribute	Levels in Part 2A	Levels in Part 2B
Category of partner support	1. I would like to receive advice, information, and support from my partner to help me quit smoking (e.g., help with learning about substitutes for smoking).	1. I am willing to provide advice, information, and support to my partner to help my partner quit smoking (e.g., help with learning about substitutes for smoking).
	2. I would like to hear expressions of empathy, love, trust, and caring (listening ears) from my partner to help me quit smoking.	2. I am willing to provide expressions of empathy, love, trust, and caring (listening ears) to help my partner quit smoking.
Positive versus negative support	1. Positive support from my partner helps me more to quit smoking (e.g., complimenting me on my efforts in smoking cessation).	1. Positive support helps my partner more to quit smoking (e.g., complimenting the partner’s efforts in smoking cessation).
	2. Negative support from my partner helps me more to quit smoking (e.g., criticizing my smoking, refusing to let me smoke in the house).	2. Negative support helps my partner more to quit smoking (e.g., criticizing my partner’s smoking, refusing to let the partner smoke in the house).
Decision-making	1. I want to have open discussions with my partner about smoking cessation and make decisions through these discussions.	1. I want to have open discussions about my partner’s smoking cessation and make decisions through these discussions.
	2. I do not want to discuss smoking cessation with my partner; I would rather discuss smoking cessation with my friends or close others.	2. I do not want to be involved in discussions about my partner’s smoking cessation; I would rather my partner discuss it with friends or close others.
Presence of partner involvement	1. I don’t want my partner involved in my quitting; that is totally up to me.	1. I don’t want to be involved in my partner’s quitting; that is totally up to him/her.
	2. I would like to have my partner involved in my quitting for support.	2. I would like to be involved in my partner’s quitting to support the change.
Strategies versus habits	1. I would like my partner to learn new strategies to support my quitting.	1. I am willing to learn new strategies for supporting my partner in quitting.
	2. I would like my partner, if I ask, to change certain habits that will help me quit.	2. I am willing to change certain habits that will help my partner quit if he/she asks.
Frequency of partner support	1. I like to receive partner support once every other day.	1. I like to provide support once every other day.
	2. I like to receive partner support one or two times a day.	2. I like to provide support one or two times a day.

**Table 3.** Preliminary discrete choice experiment findings regarding preferences for couple-based interventions and partner support

Attributes and levels	Chi-square value	*p*-value
*Format and content* of couple-based interventions (Part 1)		
Convenience	3.152	.076
Need to travel		
No need to travel		
Delivery method	0.186	.666
In-person counseling		
Website or text		
Partner involvement	7.976	.005
One intervention^†^		
Two interventions		
Topics	1.522	.217
Health outcomes		
Nicotine replacement		
Duration and frequency of treatment	1.094	.296
Weekly 30-minute sessions		
Monthly 1-hour sessions		
Interaction style	8.775	.003
One-way		
Two-way^†^		
*Partner support *desired by primary smokers (Part 2A)		
Category of partner support	3.998	.046
Advice, information, service		
Empathy, love, trust^†^		
Positive versus negative partner support	79.688	<.001
Positive^†^		
Negative		
Decision-making	18.761	<.001
Open discussion with partner^†^		
Open discussion with friends/close others		
Partner involvement	37.323	<.001
Involved^†^		
Not involved		
Strategies versus habits	0.039	.843
Learn new strategies		
Give up certain habits		
Frequency of partner support	0.060	.806
Once or twice a day		
Once every other day		
*Partner support *partners were willing to provide (Part 2B)		
Category of partner support	2.917	.088
Advice, information, service		
Empathy, love, trust		
Positive versus negative partner support	92.748	<.001
Positive^†^		
Negative		
Decision-making	21.909	<.001
Open discussion with partner^†^		
Open discussion with friends/close others		
Partner involvement	44.856	<.001
Involved^†^		
Not involved		
Strategies versus habits	0.080	.777
Learn new strategies		
Give up certain habits		
Frequency of partner support	2.583	.108
Once or twice a day		
Once every other day		

† Preferred option.
